# Supplementary material for: An Effective Primary Head and Neck Squamous Cell Carcinoma In Vitro Model
Source: Cells. 2019 Jun 7;8(6):555. doi: 10.3390/cells8060555 (PMC6628367; doi:10.3390/cells8060555)
Supplement: Supplementary file 1 [file cells-08-00555-s001.zip › Supplementary material_Oppel_et_al.pdf]

Supplementary material

Figure S1: Primary HNSCC cell culture of patient sample S18 in DMEM-medium containing 10% FBS.

Figure S2: Epithelial tumor cells of S4 expanding in PNEU-medium.

Figure S3: Figure S3: Marker expression of S12 and S15 cells cultured in three different media.

Figure S4: Multinuclear cells co-expressing epithelial and mesenchymal markers detected by indirect immunofluorescence.

Figure S5: CK14 and Vimentin (VIM) expression in mixed cell cultures composed of epithelial and mesenchymal populations visualized by indirect immunofluorescence.

Figure S6: CK19 and α-SMA expression in mixed cell cultures composed of epithelial and mesenchymal populations visualized by indirect immunofluorescence.

Table S1: Biological data of patient-derived tumor tissue.
(See separate document)

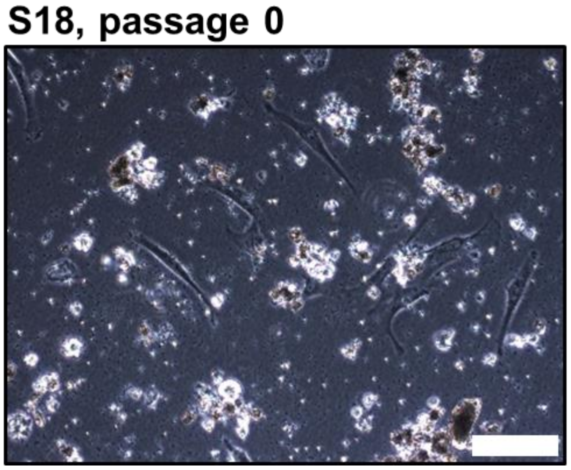

Figure S1: Primary HNSCC cell culture of patient sample S18 in DMEM-medium containing 10% FBS. Single fibroblast-like cells of S18 attach to the bottom of the cell culture dish; scale bar = 100 µm.

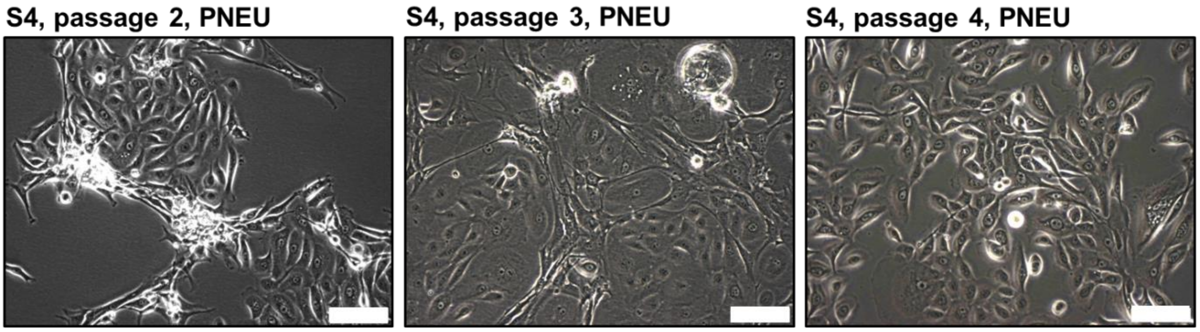

Figure S2: Epithelial tumor cells of S4 expanding in PNEU-medium. In contrast to DMEM-medium (see Figure 1), epithelial cells are enriched in PNEU-medium between passages 2 to 4 while fibroblast-like cells are lost; scale bars = 100 µm.

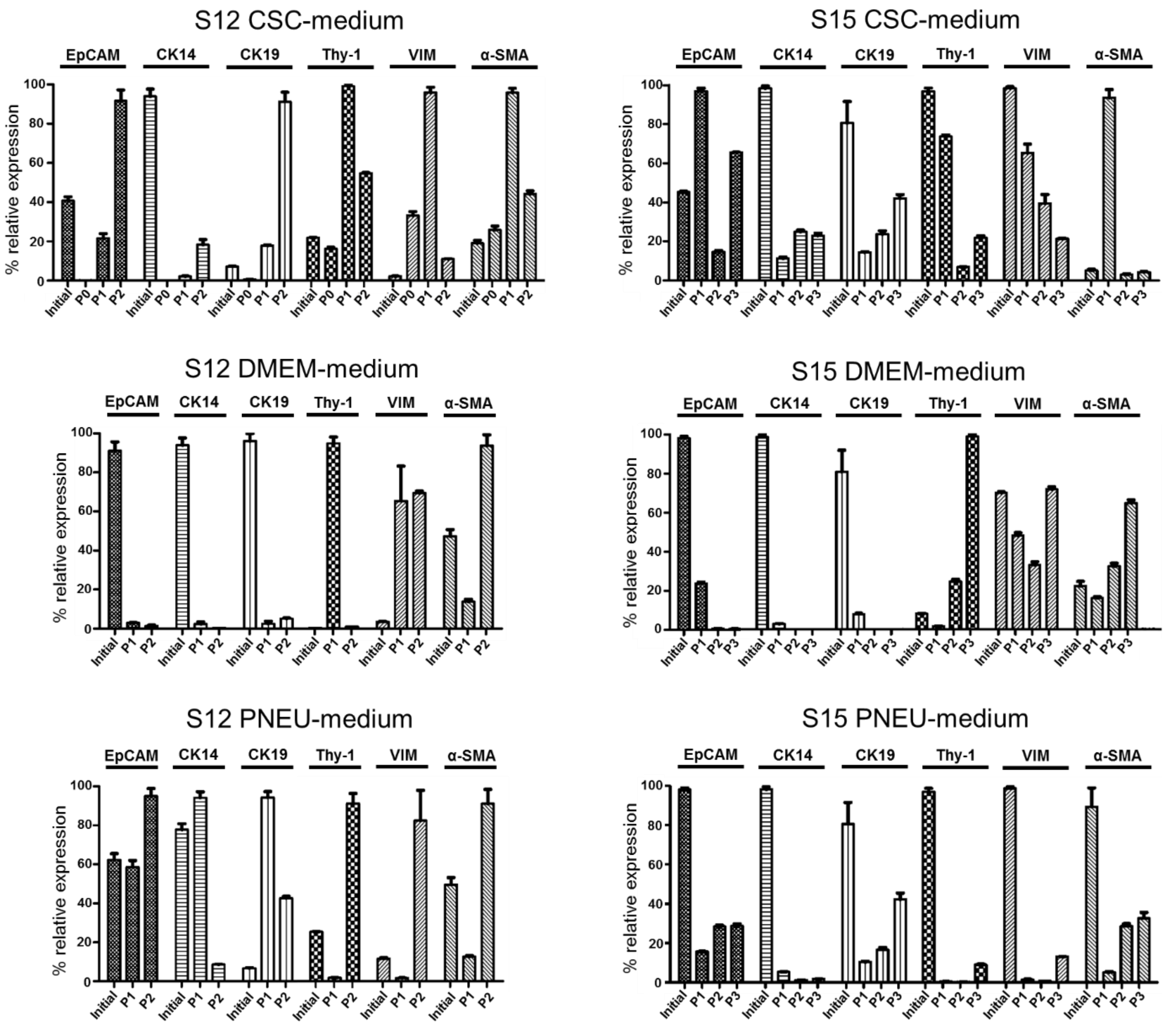

Figure S3: Marker expression of S12 and S15 cells cultured in three different media. Epithelial markers EpCAM, CK14, and CK19 and mesenchymal markers Thy-1, Vimentin (VIM), and α-SMA were analyzed by qRT-PCR; P1 = cell culture passage 1; initial = original tumor sample.

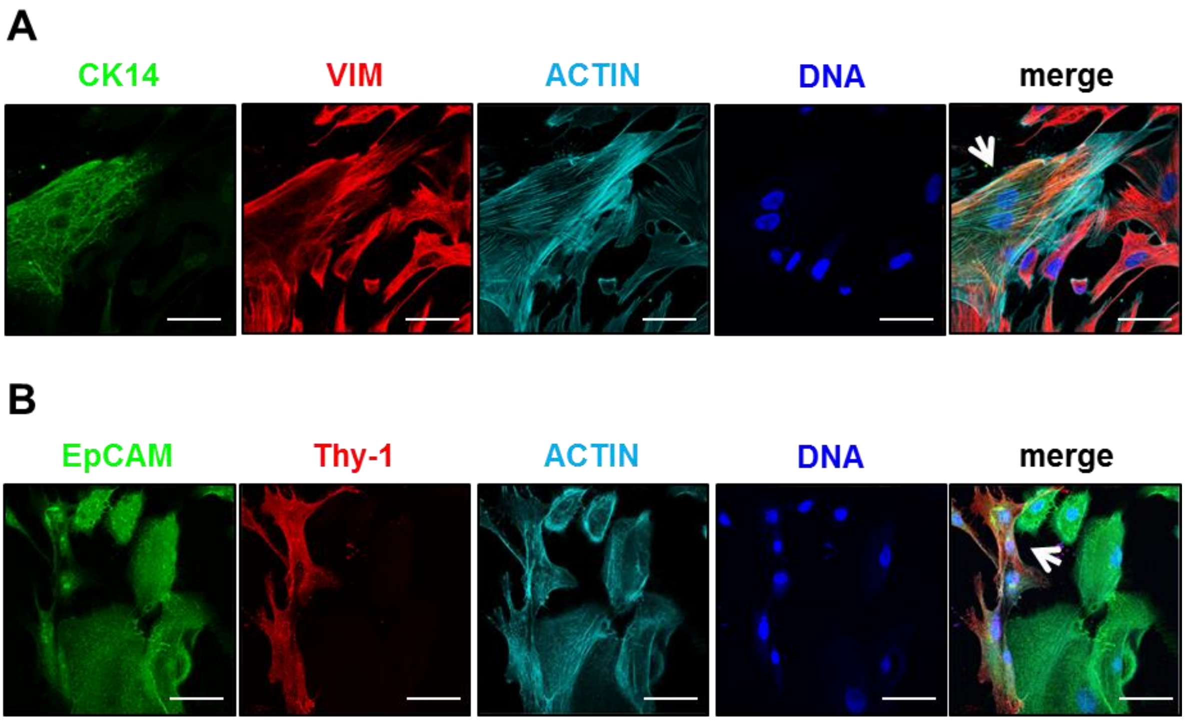

Figure S4: Multinuclear cells co-expressing epithelial and mesenchymal markers detected by indirect immunofluorescence. (A) A binuclear cell (arrow) of S22 expresses CK14 and Vimentin (VIM) and shows myofibroblast-like parallel actin fibers. **(B)** A multinuclear cell (arrow) of S4 is positive for EpCAM and Thy-1. Actin was stained using phalloidin to visualize cell morphology; DNA was stained using Hoechst33342; scale bars: 50 µm.

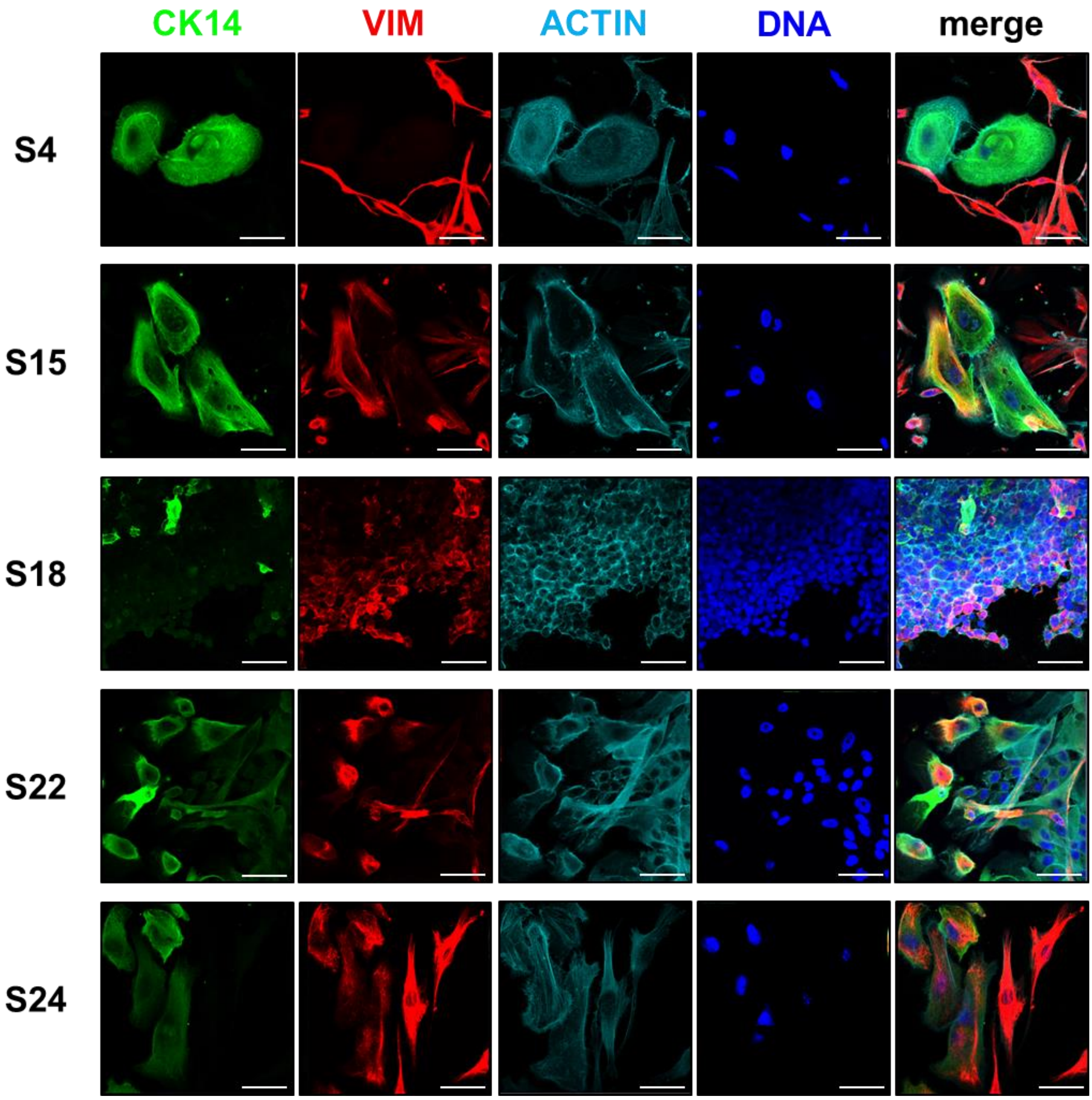

Figure S5: CK14 and Vimentin (VIM) expression in mixed cell cultures composed of epithelial and mesenchymal populations visualized by indirect immunofluorescence. CK14 and Vimentin were heterogeneously expressed in both compartments. Epithelial cells of all patients frequently co-expressed both markers, whereas cells with fibroblast-like morphology uniformly expressed Vimentin but were regularly negative for CK14. Only mesenchymal cells of S22 displayed faint CK14 staining. Actin was stained using phalloidin to visualize cell morphology; DNA was stained using Hoechst33342; scale bars: 50 µm.

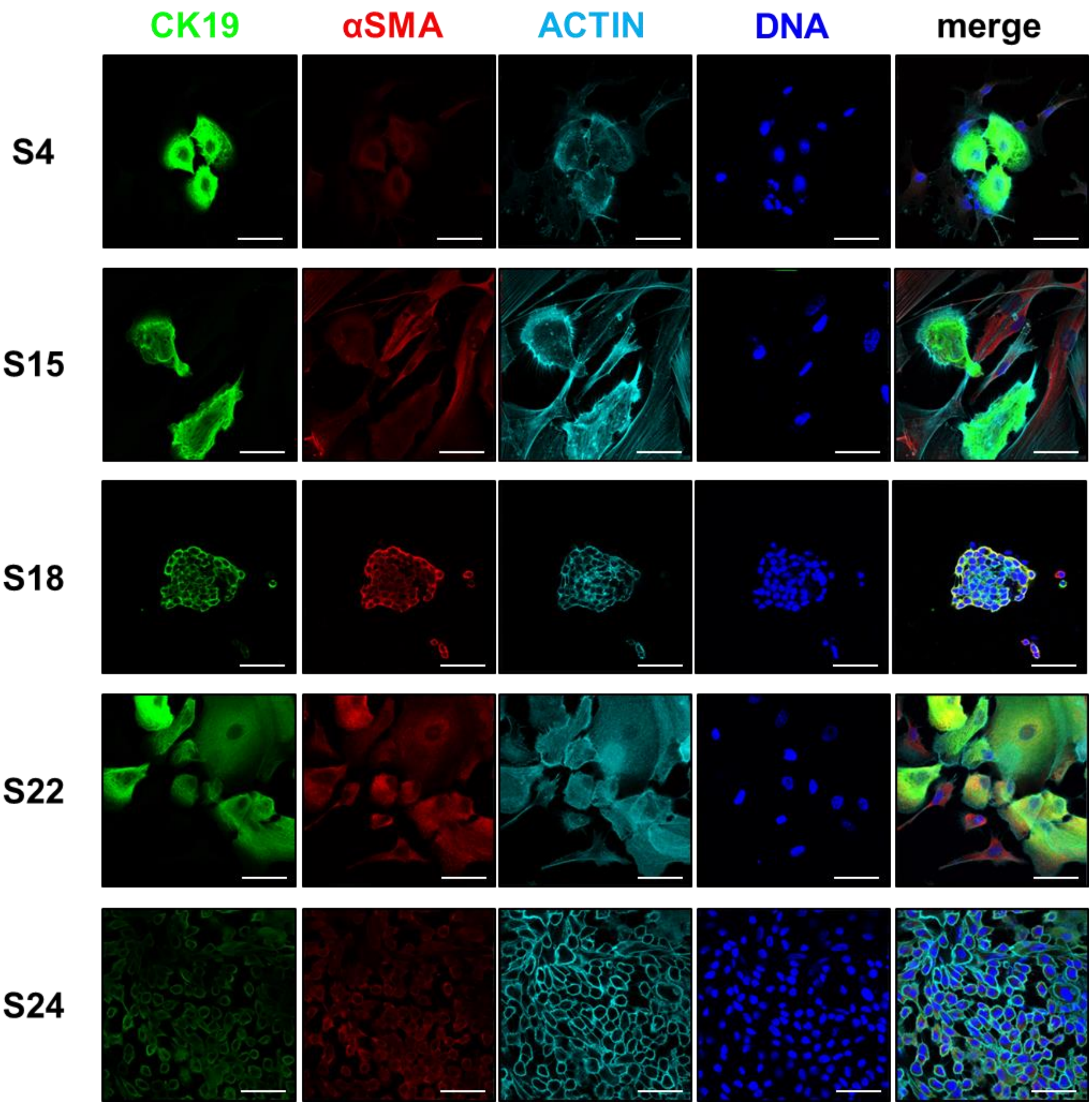

Figure S6: CK19 and α-SMA expression in mixed cell cultures composed of epithelial and mesenchymal populations visualized by indirect immunofluorescence. Epithelial cells uniformly expressed CK19, whereas α-SMA was detected in a subpopulation at varying intensity. Mesenchymal cells expressed heterogeneous levels of α-SMA. Mononuclear cells with fibroblast-like morphology showing CK19 expression were not detected. Actin was stained using phalloidin to visualize cell morphology; DNA was stained using Hoechst33342; scale bars: 50 µm.
