# Supplementary material for: An Effective Primary Head and Neck Squamous Cell Carcinoma In Vitro Model
Source: Cells. 2019 Jun 7;8(6):555. doi: 10.3390/cells8060555 (PMC6628367; doi:10.3390/cells8060555)
Supplement: Supplementary file 1 [file cells-08-00555-s001.zip › Suppl_Table_S1.pdf]

Table S1: Biological data of patient-derived tumor tissue.

Sample #	Cell culture name	Patient Sex	Patient age (years)	Tumor type	p16 status (pathology)	Differentation grade	Culture medium used	Cell culture established
S1	BI-1	male	82	Mucinous adenocarcinoma	not determined		DMEM	stroma
S2		male	78	Oropharinx SCC	positive	G3	DMEM	none
S3	BI-3	male	71	Hypopharynx SCC	positive	G3	DMEM	stroma
S4	BI-4	male	62	Larynx SCC	not determined	G2	DMEM	stroma
S5	BI-5	female	57	Oropharinx SCC	not determined	G2	DMEM	stroma
S6		male	71	Oropharinx SCC	not determined	G2	DMEM	none
S7		male	85	Oropharinx SCC	positive	G2	DMEM	none
S8		female	87	Larynx SCC	not determined	G2	DMEM	none
S9	BI-9	male	53	Schwannoma	not determined		DMEM	mesenchymal
S10		female	53	Nasal cavity SCC	not determined	G2	DMEM	none
S11		male	64	Oropharinx SCC	not determined	G2	DMEM	contaminated
S12	BI-12	male	49	Tonsil SCC	positive	G3	CSC	epithelial
S13		female	59	Larynx SCC	not determined	G2	CSC	none
S14		male	76	Tonsil SCC	positive	G3	CSC	none
S15	BI-15	female	57	Oropharynx SCC	positive	G3	CSC	epithelial
S16		female	55	Oropharynx SCC	negative	G3	CSC	contaminated
S17		female	88	Larynx SCC	not determined	G2	CSC	none
S18	BI-18	male	77	Oropharynx SCC	negative	G3	DMEM, CSC, and PNEU	epithelial (PNEU)
S19		male	67	Oropharynx SCC	positive	G2	DMEM and PNEU	none
S20		female	74	Oropharynx SCC	not determined	G2	PNEU	none
S21		female	47	Esthesioneuroblastoma	not determined		PNEU	none
S22	BI-22	male	55	Oropharynx SCC	negative	G3	PNEU	epithelial
S23	BI-23	female	88	Larynx SCC	not determined	G2	PNEU	epithelial
S24	BI-24	male	58	Oropharynx SCC	positive	G2	DMEM and PNEU	stroma (DMEM) and epithelial (PNEU)
S25	BI-25	male	75	SCC of ear/skin	not determined	G3	PNEU	epithelial
